# Modelling Animal Group Fission Using Social Network Dynamics

**DOI:** 10.1371/journal.pone.0097813

**Published:** 2014-05-15

**Authors:** Cédric Sueur, Anaïs Maire

**Affiliations:** 1 Centre National de la Recherche Scientifique, Département Ecologie, Physiologie et Ethologie, Strasbourg, France; 2 Université de Strasbourg, Institut Pluridisciplinaire Hubert Curien, Strasbourg, France; 3 Unit of Social Ecology, CP231, Université libre de Bruxelles, Campus Plaine, Brussels, Belgium; University Toulouse 1 Capitole, France

## Abstract

Group life involves both advantages and disadvantages, meaning that individuals have to compromise between their nutritional needs and their social links. When a compromise is impossible, the group splits in order to reduce conflict of interests and favour positive social interactions between its members. In this study we built a dynamic model of social networks to represent a succession of temporary fissions involving a change in social relations that could potentially lead to irreversible group fission (i.e. no more group fusion). This is the first study that assesses how a social network changes according to group fission-fusion dynamics. We built a model that was based on different parameters: the group size, the influence of nutritional needs compared to social needs, and the changes in the social network after a temporary fission. The results obtained from this theoretical data indicate how the percentage of social relation transfer, the number of individuals and the relative importance of nutritional requirements and social links influence the average number of days before irreversible fission occurs. The greater the nutritional needs and the higher the transfer of social relations during temporary fission, the fewer days will be observed before an irreversible fission. It is crucial to bridge the gap between the individual and the population level if we hope to understand how simple, local interactions may drive ecological systems.

## Introduction

Animal population dynamics directly affect the transmission of genes, illnesses and information between any individuals within a species [1], [2]. Indeed, the flow of individuals between groups and populations can have an impact on the genetic diversity of a species, thus affecting its very survival. In the same way, information flow can have a great impact on the cultural diversity of groups or the transmission of cultures between them [3]. Dispersal occurs when an individual leaves its home range to set up life elsewhere, and it is one of the mechanisms playing a role in the transmission of genes and information [4]. In species that live in social groups [5], an additional mechanism known as fission allows the dispersion of genes and information between populations. Fissions are any processes that lead to the separation of a group into several sub-groups, and they play an extremely important role in population dynamics in social species [4]. After a period of stability, fission can result in the creation of new reproductive groups, the separation of bloodlines, and the dispersion or arrival of males within a group [6].

In the case described above, the fissions are said to be irreversible (the groups that have split do not merge again). However, these fissions can also be temporary in social systems known as fission-fusion societies [7]–[9]. Factors leading to a fission are varied and range from an increase in group size to variations in the ecological environment or conflicts of interest about food resources [9], [10]. These issues are all linked to conflicts of interest when an individual chooses between staying in the group or satisfying its nutritional requirements [11], [12]. Indeed, group living in social species involves both advantages and disadvantages, whatever the level of sociality may be. An individual within a large group has less risk of predation: an individual surrounded by other group members is less likely to be attacked by a predator, and the number of individuals provides better vigilance against attacks [5]. Although group living improves generally foraging for food and reproductive success, it also has disadvantages: a larger group is more visible to predators, and increases conflicts of interest for food within the group. In order to maintain group cohesion, each individual therefore has to make a compromise between its own needs and the advantages of group living – namely its social needs [13]. When this compromise is no longer possible, i.e. one or several individuals cannot satisfy their own needs within their social group, individual emigration or an irreversible group fission can be observed [10]. Fission is often described as a response to excessive competition for resources within the group, but can also occur when the incompatible needs of each individual lead to high conflicts of interest between group members [10].

Fission results in the creation of several sub-groups, which can be temporary or permanent. The first case is that of temporary fission, or fission-fusion dynamics [8]. In the African elephant (*Loxodonta africana*), groups can be classified according to their level of cohesion [14]. This is a multi-level social structure of fission-fusion in which the most cohesive groups remain together all year. They forage together when the season permits, showing the importance of food competition and information sharing. In the hyena (*Crocuta crocuta*), a species with a fission-fusion system, the individuals form clans that constantly change due to conflicts of interest, similar physiological needs or risks of infanticide [15]. Bechstein's bats (*Myotis bechsteinii*) are capable of maintaining social relations between subgroups despite the very high fission-fusion dynamics of these groups [16], [17]. Stability in groups is the *sine qua non* for the sharing information about roosting. In elephants and bats, these temporary fissions are characterised by the fact that there very strong links already exist between all the individuals in each subgroup, and these subgroups maintain a certain global social cohesion [16], [18], [19] through the importance of one or several key individuals [20], [21]. Sometimes this cohesion is impossible, and in these cases we refer to « irreversible fission ». Irreversible fission can occur after a number of temporary fissions. A model based on spider monkeys (*Ateles geoffroyi*) showed that the links between individuals could be reinforced by the time they spent foraging together. Indeed, in an environment made up of patches of food, individuals that had foraged together more often have stronger links than those who had only met on rare occasions. The social network obtained from simulations is similar to the one described in this spider monkey study [22]. The development of preferred associations according to similar nutrient requirements has also been observed in fish [23], [24]. In the aforementioned examples, authors suggested that group members develop preferred associations because they shared similar requirements, but it is also true that animals can feed in the same patches because they are strongly associated. There is therefore a link between nutritional needs and social relationships, and this link should make it possible to predict sub-grouping patterns after irreversible fission.

Fission seems to be mainly affected by the nutritional requirements of an individual and its social links with its conspecifics [4], [7], [9], [15], [22], [25], [26]. Group size increase or environmental changes are the main issues affecting conflicts of interest for food [6], [10], [26], [27]. When it is no longer possible for an individual to meet its own needs, it will probably leave the group for a new area where it can feed. This is the case in most subsocial or social species but is not seen in eusocial species. If this necessity to leave a zone also applies to other individuals, they will move collectively as a subgroup to the new area. Individuals will therefore move according to their own needs, but their choice will be influenced by the strength of their social relations with any individuals that have already left in one direction or another [10], [22], [25]–[27]. Individuals in bonded social species (in primates and some other mammals) face a dilemma between pursuing short term nutritional interests (by dispersing) and staying with preferred conspecifics at some nutritional cost (i.e. social viscosity [28]). The social network, i.e. the social relations between members of a group taken as a whole, therefore has a strong influence on any choices and compromises (meeting nutritional requirements *vs.* social needs) that individuals will have to make [9], [15], [25], [27], [29].

It is difficult to observe fissions in the natural environment, and they cannot be reproduced experimentally in order to understand the underlying mechanisms. A social group can remain cohesive for anywhere between a few to dozens of years, and irreversible fission can last from a few months to a maximum of two years (see table 1 for references). Dittus [30] calculated that one group has a probability to split every 74 years in Toque macaques (*Macaca sinica*). This phenomenon can be studied via the use of a multi-agent system to reproduce irreversible fission and test different hypotheses. In this study, the model depends on three different hypotheses: 1) Temporary fissions and sub-group compositions should depend on nutritional needs; 2) these temporary fissions should affect the social network and result in group clusterisation; 3) the group should split irreversibly when group clusterisation is too strong, because individuals of one sub-group no longer have social relationships with individuals in the other sub-group. The mechanisms implemented in the model of this current study to simulate irreversible fission are based on the probability that an individual will go to a specific resource location. This probability depends both on the nutritional requirements of the individual and the relations this individual has with individuals that have already left for each resource area within the model [25]. In order to understand how temporary fission can modify social networks of individuals to such an extent that irreversible fission occurs, the model reproduces fission-fusion dynamics in which individuals spend their day foraging and interacting, and group together at night in a resting area [12]. This process has already been observed in lemurs (*Lemur catta*), which continued to group together for the night during the first period of group fission [31]. In our model, we tested different parameters: 1) The group size, from 10 to 20 individuals, 2) the importance of nutritional requirement compared to group cohesion and 3) the impact of temporary fissions on group social network (from weak to strong impact). The two last variables should have a negative influence on the number of days before irreversible fission is observed. Indeed, the higher the nutritional needs of group members and the higher the impact of temporary fissions on the social network, the quicker an irreversible fission will be observed.

**Table 1:** Data for observed irreversible group fissions.

n°	Common name	Latin name	Time taken for irreversible fission	Number of individuals (number in each sub-group)	Reference
1	Lemurs	*Lemur catta*	6 months	37 (22/15)	[31]
2	Rhesus macaque	*Macaca mulatta*	9 months	28 (18/10)	[43]
3	Barbary macaque	*Macaca sylvanus*	1 year	87 (50/24/13)	[6]
4	Moor macaque	*Macaca maura*	10 months	43 (26/17)	[45]
5	Barbary macaque	*Macaca sylvanus*	9 months	131 (94/37)	[61]
6	Japanese macaque	*Macaca fuscata yakui*	10 months	54 (21/33)	[44]
7	Japanese macaque	*Macaca fuscata*	2 years	64 (22/15/27)	[29]
8a	"Alto's group" baboons		2 years	24 (4/14/6)	
8b	"Hook's group" baboons		6 months	16 (7/9)	
8c	"Lodge group" baboons	*Papio cynocephalus*	1 month	27 (17/10)	[27]
8d	"Dotty's group" baboons		1 year	16 (7/9)	

## Materials and Methods

### Model

This section describes the model according to ODD Protocol (i.e., Overview, Design concepts and Details) [32], [33].

#### Purpose

The aim of this model is to study how 1) the nutritional needs of individuals and 2) group cohesion (i.e. social viscosity) can create temporary fissions, which can lead to a permanent fission via changes in the social network. The model includes three major independent variables: 1) the percentage of social relations transfer at each fission (transfer from individuals that are not in the same sub-group to individuals in the same sub-group), 2) the nutritional requirements/social needs ratio, a scaling constant defining to what extent an individual will either follow its own motivation or copy other individuals according to conflicts of interest for food resources or the heterogeneity of food requirements within the group and 3) the group size (10 or 20 individuals per group). The first two variables should have a negative impact on the number of days before irreversible fission is observed: the higher the social relation transfers and the nutritional requirements/social needs ratio are, the smaller the number of days will be before fission occurs.

#### Entities, State variables and scale

We refer to temporary fission when individuals form two separate groups during the day, but significant links are still observed between the individuals of both subgroups. We consider irreversible fission to have occurred when individuals form two separate groups, each made up of at least four individuals. If there are three or less individuals in a group (female plus juveniles or young males), this is considered to be a dispersion rather than a fission [4], [27]. Irreversible group fission happens when each individual attributes 95% of its social time to the individuals in the same area. Five percent of its time is still attributed to individuals from the other subgroup, but this is considered to be insignificant and fission is therefore irreversible [10]. In natural groups, irreversible fissions are seen to occur over periods ranging from 3 months to 2 years. In this model, we consider that a « viable » irreversible fission occurs between 90 and 900 steps (days), based on previous research (see Table 1). The source code used to implement the model can be found in Source Code S1.

The environment is composed of three areas: a resting area in which individuals remain together, and two resource areas to which individuals move depending on their needs and their social links. It is within these two last areas that links between individuals will either decrease or increase according to the individuals that are in the same area. This condition – two resource areas – limits the individuals to two decision spaces. However, it fits with our research topic since formal groups in all but one of the studies we cited (see table 1) split into just two new groups and no more. Individuals each have an identity to make it possible to differentiate between them. They are characterised by a type of activity according to the area they are in, by the sum of the links with individuals in the same resource area as them and by the nutritional requirements representing the intrinsic probability of each individual going to one resource area or the other. Each individual has a probability for each resource area. This probability might depend on the quality of the food patch [34] or the competition they experience there [35] (not included in the current model). A link (i.e. social relation) represents the time spent by one individual to groom another [10]. Each link connects two individuals and its weight represents the social time used by one individual to groom another. The links are directional, meaning that the strength of a link between individual *i* and individual *k* is not necessarily identical to the strength of the link between *k* and *i*. This condition suggested that animals are able to remember the link strength that they have with each group member. However, social animals such as primates are known to have high cognitive abilities and good social or spatial memory [36]–[38]. Bats are also able to show a good spatial memory and to maintain long-term relationships [16].

The network of social relations used in this model is an equal (egalitarian) or complete social network [39], [40]. Each individual has an allotted amount of social time (often considered to be used for grooming), and equally divides this time between the other members of the group [10], [39]. The best social network to represent the structures observed in groups of animals is a Erdos-Renyi or scale-free network [41], [42]. However, the equal network gives an initial idea of the fission phenomenon and makes its study possible. Additionally, the interest of a model lies in its ability to be more explicit, simpler and easier to use than the reality it is supposed to represent. In this way, using an equal network as a seed network is better than using a random network to understand fission-fusion dynamics and feedback loops between fission-fusion and social network. Our main aim is to understand the network evolution from a homogeneous state.

#### Process overview and scheduling

Each step of the programme represents one day. This means that there is one temporary fission per day. In the wild, irreversible fission is observed within an interval of three months to two years (see Table 1 for instances and references). However, according to several studies [6], [29], [31], [43]–[45] some temporary fissions (pre-fission) occur at a mean rate of one fission or less per day. In our simulations, we therefore set an upper limit of 900 days after which no irreversible fission would be expected to occur, as seen in the wild in observed groups of animals. Every day the needs and social links of each individual could lead to a temporary fission (see [25] and [10] for details on how structure of social networks may lead to group fission). The probability of this temporary fission (per day) and the resulting sub-groups depend on both the intrinsic possibility of each individual going into each resource area and their social links with the individuals that are already in these areas.

#### Design concepts

In order to represent the social network dynamics, the model includes the percentage of transfers within social relations or more precisely, a percentage of the time allotted to grooming. At each temporary fission, when all the individuals have moved to a resource area, the social network of each individual changes. The links each individual has with other individuals that are engaged in a different activity (i.e. in a different area) therefore decrease according to the percentage of transfers within social relations that has been determined by the experimenter (from 10% to 100%). The total decrease is equally redistributed amongst the group members carrying out the same activity as the individual (within the same area). The individual decisions made every day can lead to a redistribution of social time for each individual, according to the individuals it was in contact with the previous day. This creates a dynamic model, and the decision made by individuals to go to one resource area or another changes from one day to the next according to the decisions made on previous days. These choices will alter the relations within each dyad of individuals, affecting the social network and therefore influencing the choices made by each individual.

#### Emergence

The sequence of events in the model are presented in Figure S1.

Every day, each individual moves towards one resource area or the other. At the beginning of each movement, a random function attributes each individual with an intrinsic probability for each resource area. These two probabilities, named *λ_i,1_* and *λ_i,2_*, correspond to the individual's needs. The probability *P_i,s_* that an individual *i* will go to area *s* is the following:
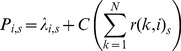
(1)where *λ_i,s_* is the intrinsic probability of individual *i* to move to area *s*, *C* is a mimetic coefficient (similar to social viscosity; [25], [28], [46]) and *Σ r(k,i)_s_* is the sum of all the links between individual *i* and individuals *k* already present in area *s*. This mimetic coefficient represents the probability to follow individuals to a resource area, that is to say a kind of measure of social cohesion/sociality. A high mimetic coefficient means that individuals show a high group cohesion/sociality/viscosity: The higher the mimetic coefficient, the higher the Nutrition/Sociality ratio and the lower the probability is that the individual will move according to its own nutrient requirements. The artificial groups are made up of 10 or 20 individuals, a group size similar to those in empirical studies ([25], [47]–[51]). *P_i,1_* + *P_i,2_* + *P_i,0_*  =  1, where *P_i,1_* is the probability of moving to resource area s_1_, *P_i,2_* is the probability of going to resource area s_2_ and *P_i,0_* is the probability of staying in the resting area (state s_0_). In this way, *P_i,s_* ≤ 1.

To calculate the Nutrition/Sociality ratio, we proceeded on the basis that the sum of the probabilities *P_i,s_* (equation 1) of all possible actions for an individual should be equal to 1.

As we know that *P_i,0_*  =  1 – (*P_i,1_* + *P_i,2_*), then (*P_i,1_* + *P_i,2_)* ≤ 1.

In this model, conflict of interests are based on the two states (i.e. the resource areas s1 and s2). Therefore, in order for individual *i* to leave the resting area:

(Σ r(k,i)_1_ + Σ r(k,i)_2_)  =  100, therefore (λ_i,s1_ + λ_i,s2_) ≤ (1 - C * 100).

The Nutrition/Sociality ratio (Table 2) is calculated as follows: R  =  (λ_i,1_ + λ_ i,2_)/(C * 100)

**Table 2:** Detail of the calculation of the different ratios used in the theoretical model.

Mimetic coefficient	λ_1_	λ_2_	Nutrition/Sociality Ratio
0.001	0.45	0.45	9
0.002	0.4	0.4	4
0.003	0.35	0.35	2.33
0.004	0.3	0.3	1.5
0.005	0.25	0.25	1
0.006	0.2	0.2	0.67
0.007	0.15	0.15	0.43
0.008	0.1	0.1	0.25
0.009	0.05	0.05	0.11

#### Stochasticity

This model is stochastic. At each step, a random number *x* ranging between 0 and 1 is generated for each individual. This number determines whether an individual moves from state s_0_ (resting area) to state s_1_ (resource area s_1_) or state s_2_ (resource area s_2_), according to the possibilities of moving to each of these two states. If P_i,1_ ≥ x, the individual is moving towards s1. If (P_i,1_ + P_i,2_) ≥ x > P_i,1_, then the individual is moving towards s2. If (*P_i,1_ + P_i,2_*) < x, the individual remains in the resting area. This procedure is carried out for each individual in area 0 until all the individuals have moved to one of the two resource areas.

#### Interaction

When all the individuals have moved, they form one group (in one resource area) or two groups (one in each of the two areas), and their behaviours hence result in either cohesion or fission. If temporary fission occurs, the links between individuals in one same area will be reinforced whilst the links between individuals within a different area will decrease by a percentage of social relations transfer, imposed by the experimenter. At the end of the day in question, all individuals return to the resting area. If irreversible fission occurs, the simulation is stopped.

#### Collectives

When all the individuals have moved to a resource area, the social network of each individual changes. The link *r(k,i)* each individual *i* has with other individuals engaged in a different activity (i.e. in a different area *s*) therefore decreases according to the percentage of social relations transfer determined by the experimenter (from 10 to 100%). The total of these decreases is equally redistributed amongst group members carrying out the same activity as the individual (within the same area). If individual *i* is alone in an area, its social network (i.e. links from *i* to *k*) is not modified, but the links between the other individuals and this single individual (i.e. the links from *k* to *i*) will be changed. We formulated the change of the link *r(k,i)* as:
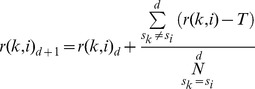
(2)where *d* is the day of the fission and *d+1* the next day, T is the percentage of social relations transfer, *s* is the state of individuals and N is the number of individuals *k* in the same state as *i* at day *d.*

#### Observation

If the programme runs through to the maximum 900 steps, we consider that fission has not occurred. We tested each Nutrition/Sociality ratio for every transfer percentage (in ten equal steps from 10 to 100%) in groups of 10 and 20 individuals. We carried out 1 000 simulations to obtain the number of steps ( = number of days) before irreversible fission occurred (main dependent variable) for each set of parameters tested (number of individuals, nutritional requirements, mimetism and percentage of social relations transfer).

#### Initialisation

At the beginning of each simulation, the individuals are in the resting area. The social structure is identical at the beginning of each simulation. The social network is considered to be equal, meaning that all individuals attribute equal amounts of their grooming time to each of the other group members. Individuals have 100% of their grooming time, meaning that when the simulation initiates, the amount of time attributed to grooming another individual is therefore 100/(n−1), where n is the number of individuals in the group. This amount of time will vary according to the temporary fissions that could occur during the simulation, but the sum of an individual's links with the other members of the group, whatever their activity, will always be 100.

#### Input

The model was developed in Netlogo 3.15 [40], [47], [52]. One time step in the simulation represents one day. The model includes three major independent variables: 1) the percentage of social relations transfer at each fission (transfer from individuals that are not in the same sub-group to individuals in the same sub-group), 2) the nutritional requirements/social needs ratio, a scaling constant defining to what extent an individual will either follow its own motivation or copy other individuals according to conflicts of interest for food resources or the heterogeneity of food requirements within the group and 3) the group size (10 or 20 individuals per group).

### Statistical analyses

For each simulation, we obtained a number of steps ( = number of days) that depended on three independent variables: the Nutrition/Sociality ratio, the percentage of social relations transfer and the number of individuals (N =  10 or N = 20 individuals).

We used a generalised linear model (GzLM) for all the data in order to determine the influence of the independent variables on the dependent variable. The GzLM is used to study data with non-normal distribution and followed in this study the Poisson law (used when variables are discrete). Data obtained from 900 simulations were used for this test. Once this was done, we examined the relation between the percentage of social relations transfer and the mean number of days before the observation of irreversible fission. A survival curve was used to establish the type of relation between these two variables (linear, logarithmic or exponential), then we carried out a Kruskal-Wallis test followed by Dunn's multiple comparison test to study the relation between the Nutrition/Sociality ratio and the number of days before irreversible fission. Simulations at 10% transfer were not included in these tests in order to reveal the longest periods observed below the 900-day threshold.

Analysis was carried out using R 2.12.0 software [53]. The significance threshold for all these tests was fixed at 0.05.

## Results

**Figure 1:**
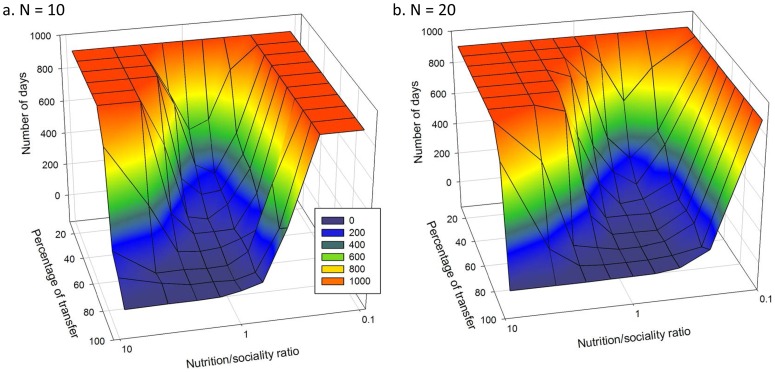
The influence of the Nutrition/Sociality and the percentage of social relation transfer on the average number of days before irreversible fission is observed for (a.) N = 10 individuals and (b.) N = 20 individuals.

We first sought to understand which values of the two independent variables (percentage of social relations transfer and the Nutrition/Sociality ratio) influenced the number of days leading to irreversible fission in artificial groups. Figure 1 shows the field of values for the two variables in which irreversible fission is observed between days 90 and 900, the period during which fission is considered to be observable in groups of non-human primates (see table 1 for references). Overall, group fission probability has the same profile for groups of 10 or 20 individuals. A generalised linear model (GzLM) was used for all the data and showed that the independent variables - namely the group size, the Nutrition/Sociality ratio and the percentage of social relations transfer - had an effect on the dependent variable, i.e. the number of days leading to a fission (AIC  =  265573; 899 degrees of freedom; Fig.1). The group size has a negative influence on the number of days (z = −3,246, p =  0.00117), meaning that the greater the increase in the group size, the sooner fission will occur. The percentage of social relations transfer also shows a negative effect, namely that the number of days leading to fission decreases as the percentage of social relations transfer increases (z = −292, p<0.0001). The relation between the two variables is linear for both group sizes, N = 10 (r^2^ = 0.97, F_1,8_ = 280, p<0.0001) and N = 20 (r^2^ = 0.99, F_1,8_ = 721, p<0.0001). The Nutrition/Sociality ratio had a positive effect on the number of days: the number of days increases with the increased ratio, and also therefore with the increased nutritional requirements in relation to social needs (z = 99,674, p<0.0001). A high Nutrition/Sociality ratio reflects high needs (or different needs in a heterogeneous group) or low group cohesion.

**Figure 2:**
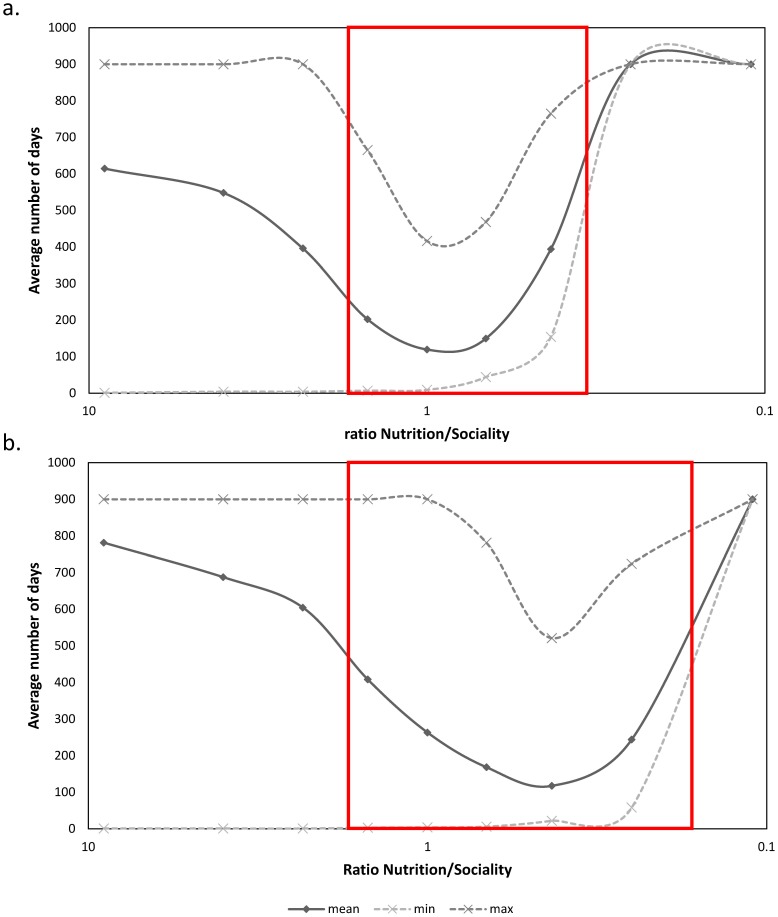
Average (continuous line), minimal and maximal (dotted line) number of days before irreversible fission is observed according to the Nutrition/Sociality ratio (logarithmic scale; including all transfer percentages from 20% to 100%) for (a.) N = 10 individuals and (b.) N = 20 individuals. For simulations where the social relations transfer percentage is 10%, no fission was observed as the programme continues to a threshold of 900 steps. Simulated data inside the red rectangle are coherent with observed data in non-human primate groups.

Analysis has revealed that globally, the Nutrition/Sociality ratio positively influences the number of days before irreversible fission is observed. However, figure 2 shows that this influence is not linear but follows a U-shape. A Kruskal-Wallis test followed by Dunn's multiple comparison test confirmed this for groups of both 10 (N = 9; K = 169; P<0.0001) and 20 individuals (N = 9; K = 147; P<0.0001). Detailed results of the Dunn's test can be found in table S1. Whatever the number of individuals in the group (N  =  10 or N =  20), 3 different value groups become apparent for the Nutrition/Sociality ratio. A first group of values below 0.43 for N = 10 and 0.25 for N = 20 is observed from day 90–900 and corresponds to situations where no fission occurs because group cohesion is too high. Next, there is a group with values varying between the aforementioned values and 2.33, showing fissions observed for relatively low « number of days » values. Finally, there is a group of Nutrition/Sociality values above 2.33, where the average number of days before fission is longer.

## Discussion

**Figure 3:**
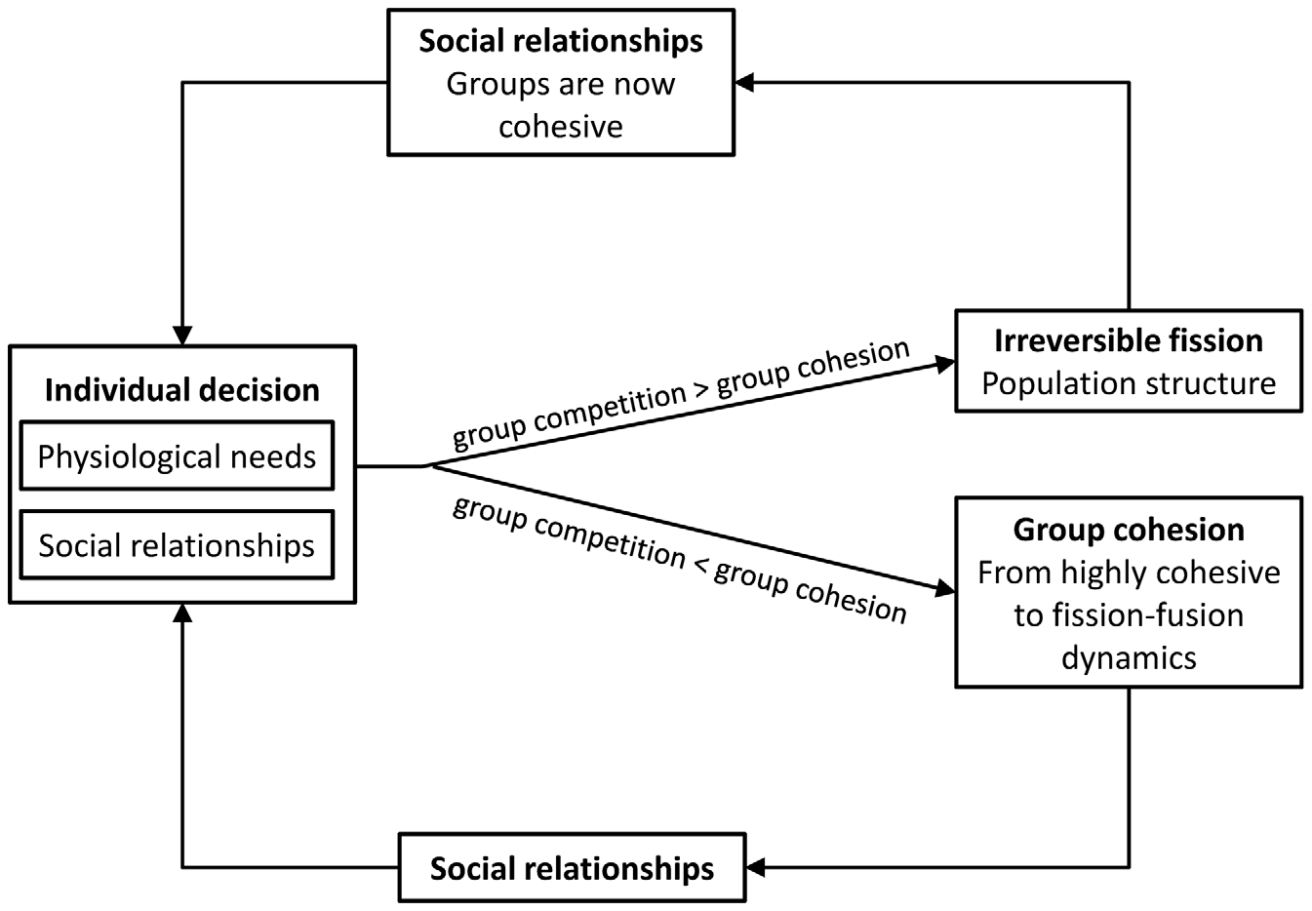
Schematic relationships between the individual decision (based on physiological needs and social relationships), the group cohesion (from highly cohesive groups to fission-fusion dynamics) and the population structure (affected by irreversible fusion). There is a feedback loop between social relationships and the group cohesion/fission-fusion dynamics that allows us to understand the dynamics of fission in animal groups.

The aim of this study was to construct a social network dynamics model capable of simulating a succession of temporary fissions that could lead to irreversible fission. This dynamic process is summarised in the figure 3. The number of days before irreversible fission occurs is shown to be influenced by many factors such as the Nutrition/Sociality ratio (nutritional requirements divided by social needs or cohesion), the percentage of social relations transfer (from individuals not in the same sub-group to individuals in the same subgroup) and the group size.

The results showed that the Nutrition/Sociality ratio can be separated into three distinct categories for its influence on the number of days before irreversible fission occurs, with the central zone corresponding to relatively fast fissions occurring within a month to a year. The central part of figure 2 (red rectangle) shows the results of this study to be coherent with the number of days observed before irreversible fission occurs in non-human primate groups. Here social links represent the propensity of each individual to show a high social dependence or sociality [18], [19]. A high Nutrition/Sociality ratio means that the individuals will give priority to their nutritional requirements by heading towards a resource area rather than preferring their social links with the other individuals already present in each zone. This might happen when conflicts of interest are high between individuals because of their different nutritional requirements. Fission occurs later, as individuals that have already reinforced their links for Day 1 will not necessarily be in the same area on days 2 or 3, depending on their nutritional requirements. A longer period is therefore necessary to reinforce social links to the extent that they can influence the decisions of each individual, and this results in a group clustering. In this case, irreversible fission is only observed in the lowest ratios when the percentage of social relations transfer is high: for N = 10 and a ratio below 1.5, no fission was observed below 50%, and for N = 20 and a ratio below 1.5, no fission was observed below 70%. In other words, a high Nutrition/Sociality ratio makes that individuals to be more independent, and weakens the mimetic mechanism. As irreversible fission can only be observed when strong links have been created within a subgroup, it cannot logically be observed when individuals are behaving in a practically independent manner.

When the Nutrition/Sociality ratio is weak, social links (i.e., group cohesion) are more important for individuals, and this is what determines their choice of area. No fission whatsoever was observed in extreme cases where the ratio was 0.11, irrespective of whether there were 10 or 20 individuals in the group. This was also the case for a ratio of 0.25 for N = 10. No fission was observed because individuals showed great cohesion and high social dependence. This means that on each successive day, the initial group will not split into two subgroups, and shows that this phenomenon does not happen often enough for us to observe sufficient modification of the social network to entail irreversible fission. This strong cohesion can be observed in the wild when ecological pressure is strong [5]. The latter can take the form of predation (group cohesion is reinforced by high predatory pressure in order to reduce the individual risks of predation; [54], [55]) or food availability (a heterogeneous environment with dispersed food resources can lead to strong cohesion according to the theories of sharing and the information centre; [9], [56]). We observed a strong phase shift in the Nutrition/Sociality ratio from 1 to 0. This shift may differentiate bonded social (i.e. cohesive) species from those showing fission-fusion dynamics, even if authors have recently been considering a gradient between the two social systems than a real differentiation [9], [57].

If our simulations reflect what happens in animal groups, i.e. if the relation between the transfer of social relations and the number of days is correctly replicated, the percentage of social relations transfer, at least in primate groups, should be high in order to result in irreversible fission. Indeed, if we trust the model, two groups (7 and 8a, see table 1) should have a social relations transfer percentage of about 20–30% per fission but most primate groups (see table 1) should have a social relations transfer percentage of over 70% per fission. This clearly contradicts the very definition of primate groups that are reputed to have stable relations over time [58]–[60] in comparison with temporary groups such as shoals of fish or flocks of birds [7]. However, these studies listed in Table 1 are obtained from primate groups during the period of irreversible fission, when social relationships are particularly unstable. This could explain why we observed these high values of percentage of social relations transfer. On the other hand, no study to date has shown the effect a temporary fission can have on the strength of social relation between two individuals or within the group as a whole, even if many studies have examined the factors (kinship, affiliation, dominance) affecting decisions of individuals and composition of new groups [27], [29], [45].

These high values of social relations transfer could be also explained by the criterion of only one temporary fission per day. If we increased this number to a minimum of two pre-fissions, then the percentage of social relations transfer should decrease along with the number of days to observe fission, and our results should be more similar to those observed in wild social species. However, previous studies claim that only one pre-fission, or even no sign of group fission at all, are observed before irreversible fission occurs [6], [29], [31], [43]–[45]. We only found two or more pre-fissions per day in Prud'Homme's study [61] of semi-free ranging Barbary macaques. Most of the observed irreversible fissions lasted for up to a year. Menard and Vallet [6] observed fission in the Barbary macaque (*Macaca sylvanus*) and considered it to last two years, yet the initial group reformed during the birth season and another fission then became irreversible. This period lasted for one year. A similar phenomenon was observed in Japanese macaques (*Macaca fuscata yakui;* [29]). In this case, the fission took two years to occur but was divided into two periods: the first concerns two groups that began a fission in September 1976 and ended in January 1978, and the second period overlapped the first and started in September 1977, ending in August 1978. A third case involves baboons [27], but data about the history of the fission in this group are limited. We have however noticed that each of these three groups split into three subgroups, whereas groups that split more quickly only produced two subgroups (see Table 1 for subgroup composition).

In our model, all the individuals in a given group have the same transfer percentage of social relations, contrary to observations in a group of Japanese macaques [44] and a group of moor macaques (*Macaca maurus;* [45]). The authors observed fission after 5 and 4 months respectively for females, whereas males attained irreversible fission after 10 months. Individuals tend to follow other individuals with whom they already have strong links. In moor macaques [45], Barbary macaques [61] or rhesus macaques (*Macaca mulatta;* [43]), matrilines are preserved in each subgroup, whilst high-ranking lemur individuals [31] (who spend their time together) will separate themselves from the low-ranking individuals. We can therefore consider that it is not too costly for an individual to « sacrifice » 70% or even 90% of its links to individuals with whom it spends little time. The fission mechanism based on nutritional requirements/social needs ratio, as used in this current model, makes it possible for a group to maintain a stable relation between the size of the group and the availability of food in the surrounding environment. When one of these parameters changes, so does the ratio, potentially resulting in either a stronger group or faster fission. It would be interesting to quantify the changes in social relations within animal groups and understand how these relations change within the natural context depending on variations in group demography or ecological environment.

In this model, we used an equal or egalitarian social network. This type of network is not found in the natural environment. So-called egalitarian societies exist [62], as described for the Tonkean macaque (*Macaca tonkeana*), but their social network is still described as random (Erdos-Renyi or scale-free, [40], [41]) even if there is a relatively homogeneous distribution of relations between individuals. An equal or egalitarian network should lead to slower fissions in our model than those seen in a random social network or in a social network where preferential associations can be observed (in dyads or even in subgroups of related or dominant individuals). This should also be checked in free-ranging groups of animals in the wild. However, the duration of fissions can be empirically similar in some groups despite their differing types of social organisation, whereas for groups of the same species with similar group sizes, fissions differing in both time and manner can be observed (see table 1). The four groups of baboons [27] illustrate this well: the four groups showed fissions within one month to two years. « Dotty's group » containing 16 individuals and « Lodge group », containing 27 individuals proved to be more influenced by kinship whilst the strong links between non-related individuals in the « Hook's group » of 16 individuals was the determining factor of subgroup composition. This study is moreover limited to the exclusive use of an egalitarian network. Other types of social networks should be implemented within this model, and it is also necessary to understand how different social relations between individuals can affect the speed at which fission occurs. A key element to validate this study is the implementation of non-human primate group social networks within the model that we have developed. This would enable us to directly compare the fissions that can be observed in the wild and in the model, not only in terms of time but also in terms of the social structure of the two resulting subgroups.

Whether it is temporary or irreversible, fission is a complex and little-understood phenomenon. Studies propose different causes such as the size of the group or conflicts of interest for food, but it is difficult to draw a general conclusion as possibilities to observe this phenomenon are rare, making it difficult to study. This study and the new model it describes make it possible to examine fission in greater detail and make it reproducible, hence enabling researchers to test different hypotheses about the mechanisms underlying group fission. A more developed model than the one used here could not only take different types of social network into account, but also consider a more complicated ecological environment, thus providing a more accurate picture of observed fission [22]. Our model only considered two available foraging patches. However, if the group had a larger number of possible foraging patches, then it would always split when social connections are weak. As a consequence, the U-shaped curve might be influenced by the limited number of options available in the simulation. This stage could provide vital information for our comprehension of fission mechanisms and hence contribute to a better understanding of population dynamics. The dynamic system of feedback loops between social network and patterns of fission-fusion could explain why in a same species, the lag over which groups split irreversibly can last from one month to several years. Whilst the fission process is the same whatever the group, the individuals' different needs and strength of social relationships lead to different durations before fission. Some other factors such as recognition and memory of social relationships may also be important in the fission patterns. Indeed, species like primates or elephants display stable and complex relationships as they have a large neocortex size [14], [63]. Bridging the gap between the individual and the population level is crucial to attain a detailed understanding of how local interactions drive population structure and ecological systems.

## Supporting Information

Figure S1 **Sequence of event of the model. Activity diagram created with ArgoUML 0.26.**
(DOC)

Source Code S1 **The source code used to implement the model.**
(ZIP)

Table S1 **Results for the Dunn multiple comparison tests for a.) groups of 10 individuals and b.) groups of 20 individuals. r means nutrition/sociality ratio.**
(DOC)
