# Supplementary figures and images for: Modelling Animal Group Fission Using Social Network Dynamics
Source: PLoS One. 2014 May 15;9(5):e97813. doi: 10.1371/journal.pone.0097813 (PMC4022680; doi:10.1371/journal.pone.0097813)

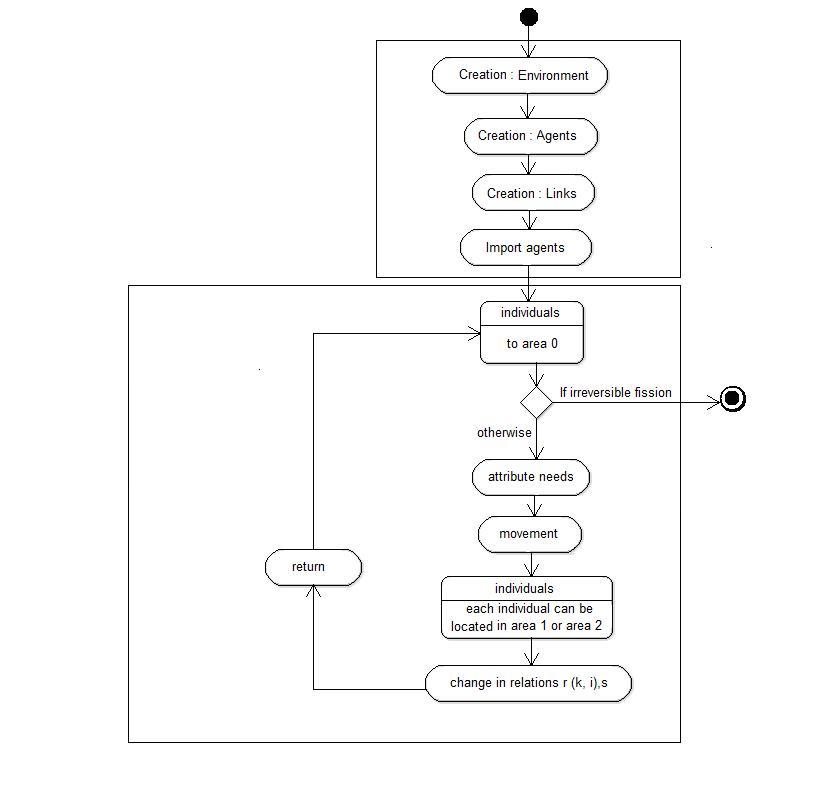

Supplement: Figure S1 — Sequence of event of the model. Activity diagram created with ArgoUML 0.26. (DOC) [file pone.0097813.s001.doc]
