# Supplementary material for: Modelling Animal Group Fission Using Social Network Dynamics
Source: PLoS One. 2014 May 15;9(5):e97813. doi: 10.1371/journal.pone.0097813 (PMC4022680; doi:10.1371/journal.pone.0097813)
Supplement: Table S1 — Results for the Dunn multiple comparison tests for a.) groups of 10 individuals and b.) groups of 20 individuals. r means nutrition/sociality ratio. (DOC) [file pone.0097813.s003.doc]

1. Groups of 10 individuals

| Dunn’s test | Rank difference | P-value |
| --- | --- | --- |
| r0.11 vs r0.25 | 0 | ns |
| r0.11 vs r0.43 | 128.9 | *** |
| r0.11 vs r0.67 | 190.7 | *** |
| r0.11 vs r1 | 213.2 | *** |
| r0.11 vs r1.5 | 197.1 | *** |
| r0.11 vs r2.33 | 146.1 | *** |
| r0.11 vs r4 | 105.2 | *** |
| r0.11 vs r9 | 80.82 | * |
| r0.25 vs r0.43 | 128.9 | *** |
| r0.25 vs r0.67 | 190.7 | *** |
| r0.25 vs r1 | 213.2 | *** |
| r0.25 vs r1.5 | 197.1 | *** |
| r0.25 vs r2.33 | 146.1 | *** |
| r0.25 vs r4 | 105.2 | *** |
| r0.25 vs r9 | 80.82 | * |
| r0.43 vs r0.67 | 61.84 | ns |
| r0.43 vs r1 | 84.29 | * |
| r0.43 vs r1.5 | 68.23 | ns |
| r0.43 vs r2.33 | 17.16 | ns |
| r0.43 vs r4 | -23.68 | ns |
| r0.43 vs r9 | -48.07 | ns |
| r0.67 vs r1 | 22.45 | ns |
| r0.67 vs r1.5 | 6.39 | ns |
| r0.67 vs r2.33 | -44.68 | ns |
| r0.67 vs r4 | -85.52 | * |
| r0.67 vs r9 | -109.9 | *** |
| r1 vs r1.5 | -16.06 | ns |
| r1 vs r2.33 | -67.13 | ns |
| r1 vs r4 | -108 | *** |
| r1 vs r9 | -132.4 | *** |
| r1.5 vs r2.33 | -51.07 | ns |
| r1.5 vs r4 | -91.91 | ** |
| r1.5 vs r9 | -116.3 | *** |
| r2.33 vs r4 | -40.84 | ns |
| r2.33 vs r9 | -65.23 | ns |
| r4 vs r9 | -24.39 | ns |

Ns : Non significant ; * : P < 0.05 ; ** : P < 0.01 ; *** : P<0.001

1. Groups of 20 individuals

| Dunn’s test | Ranks difference | P-value |
| --- | --- | --- |
| r0.11 vs r0.25 | 161.9 | *** |
| r0.11 vs r0.43 | 202.8 | *** |
| r0.11 vs r0.67 | 204.1 | *** |
| r0.11 vs r1 | 175.8 | *** |
| r0.11 vs r1.5 | 138.4 | *** |
| r0.11 vs r2.33 | 89.04 | * |
| r0.11 vs r4 | 60.16 | ns |
| r0.11 vs r9 | 38.8 | ns |
| r0.25 vs r0.43 | 40.96 | ns |
| r0.25 vs r0.67 | 42.18 | ns |
| r0.25 vs r1 | 13.94 | ns |
| r0.25 vs r1.5 | -23.48 | ns |
| r0.25 vs r2.33 | -72.84 | ns |
| r0.25 vs r4 | -101.7 | ** |
| r0.25 vs r9 | -123.1 | *** |
| r0.43 vs r0.67 | 1.22 | ns |
| r0.43 vs r1 | -27.02 | ns |
| r0.43 vs r1.5 | -64.44 | ns |
| r0.43 vs r2.33 | -113.8 | *** |
| r0.43 vs r4 | -142.7 | *** |
| r0.43 vs r9 | -164 | *** |
| r0.67 vs r1 | -28.24 | ns |
| r0.67 vs r1.5 | -65.66 | ns |
| r0.67 vs r2.33 | -115 | *** |
| r0.67 vs r4 | -143.9 | *** |
| r0.67 vs r9 | -165.3 | *** |
| r1 vs r1.5 | -37.42 | ns |
| r1 vs r2.33 | -86.78 | * |
| r1 vs r4 | -115.7 | *** |
| r1 vs r9 | -137 | *** |
| r1.5 vs r2.33 | -49.36 | ns |
| r1.5 vs r4 | -78.24 | ns |
| r1.5 vs r9 | -99.6 | ** |
| r2.33 vs r4 | -28.88 | ns |
| r2.33 vs r9 | -50.24 | ns |
| r4 vs r9 | -21.36 | ns |

Ns : non significatif ; * : P < 0.05 ; ** : P < 0.01 ; *** : P<0.001
